# Spontaneous Production Rates in Music and Speech

**DOI:** 10.3389/fpsyg.2021.611867

**Published:** 2021-05-31

**Authors:** Peter Q. Pfordresher, Emma B. Greenspon, Amy L. Friedman, Caroline Palmer

**Affiliations:** ^1^Department of Psychology, University at Buffalo, State University of New York, Buffalo, NY, United States; ^2^Department of Psychology, McGill University, Montreal, QC, Canada; ^3^Department of Psychology, Monmouth University, West Long Branch, NJ, United States

**Keywords:** tempo, music performance, endogenous rhythm, spontaneous production rates, speaking rate

## Abstract

Individuals typically produce auditory sequences, such as speech or music, at a consistent spontaneous rate or tempo. We addressed whether spontaneous rates would show patterns of convergence across the domains of music and language production when the same participants spoke sentences and performed melodic phrases on a piano. Although timing plays a critical role in both domains, different communicative and motor constraints apply in each case and so it is not clear whether music and speech would display similar timing mechanisms. We report the results of two experiments in which adult participants produced sequences from memory at a comfortable spontaneous (uncued) rate. In Experiment 1, monolingual pianists in Buffalo, New York engaged in three production tasks: speaking sentences from memory, performing short melodies from memory, and tapping isochronously. In Experiment 2, English-French bilingual pianists in Montréal, Canada produced melodies on a piano as in Experiment 1, and spoke short rhythmically-structured phrases repeatedly. Both experiments led to the same pattern of results. Participants exhibited consistent spontaneous rates within each task. People who produced one spoken phrase rapidly were likely to produce another spoken phrase rapidly. This consistency across stimuli was also found for performance of different musical melodies. In general, spontaneous rates across speech and music tasks were not correlated, whereas rates of tapping and music were correlated. Speech rates (for syllables) were faster than music rates (for tones) and speech showed a smaller range of spontaneous rates across individuals than did music or tapping rates. Taken together, these results suggest that spontaneous rate reflects cumulative influences of endogenous rhythms (in consistent self-generated rates within domain), peripheral motor constraints (in finger movements across tapping and music), and communicative goals based on the cultural transmission of auditory information (slower rates for to-be-synchronized music than for speech).

One of the most compelling questions in music cognition concerns the degree of association between cognitive functions underlying music and spoken language (Peretz and Coltheart, 2003; Patel, 2008; Zatorre and Gandour, 2008). These domains share many features, in that both involve the communication of complex auditory event sequences in which timing plays a critical role. At the same time, many salient differences characterize each domain, including the fact that the rate at which syllables are produced in speech tends to be much faster than the rate at which notes or chords are produced in music (Patel, 2014; Ding et al., 2017).^1^ The present research addresses a related issue, whether the *spontaneous production rate* (SPR) at which an individual produces speech correlates with the SPR at which that same individual produces music. Spontaneous rates in speech and music refer to rates of natural (sounded) production that are spontaneously generated by participants, and are self-sustaining in the absence of any external rate cues (such as a metronome). SPRs vary considerably across individuals within domain, but show consistency within individuals across stimuli, across hand and finger movements, and across time (for speech, see Jacewicz et al., 2009; Clopper and Smiljanic, 2011; for music, see Loehr and Palmer, 2011; Zamm et al., 2015, 2016; Schultz et al., 2016). To our knowledge, no study to date has addressed whether individual differences in spontaneous production rates are correlated across the domains of speech and music, the focus of the current study.

Different theoretical frameworks lead to different predictions regarding unique or common spontaneous rates across domains. One framework proposes that the timing of music and speech rely on a common *endogenous rhythm*, thought to be controlled by the central nervous system. Specifically, SPRs may reflect the most stable state among possible movement trajectories, that requires the least energy to produce (Hoyt and Taylor, 1981; Peelle and Davis, 2012; Poeppel and Assaneo, 2020). Spontaneous production rates may arise from a stable limit cycle oscillator; that is, a limit cycle that generates self-sustained oscillations of a constant natural frequency. Recent research suggests that spontaneous music performance rates may be based on endogenous rhythms. Musicians perform with greater temporal precision (stability) at their individual SPR than at other rates (Zamm et al., 2018), and both musicians and non-musicians synchronize their performances most accurately with auditory stimuli whose rates match their SPR (Scheurich et al., 2018). In addition, musicians with similar SPRs in solo performance exhibit better synchronization in duet performance than do partners with different solo SPRs (Zamm et al., 2016). These results are consistent with the prediction that performances at non-SPR rates yield unstable states that are more difficult to maintain accurately and precisely. The idea that speech rates reflect an endogenous rhythm is more controversial (Cummins, 2012a; Brown et al., 2017); however, several results from speech are consistent with an oscillator framework. Speech timing in a rhythmic speech cycling task suggests that speakers segment the repeated intervals consistent with an oscillator model (Cummins and Port, 1998). Speakers also time their turn-taking during conversations to match the rate of their partner (Wilson and Wilson, 2005; Schultz et al., 2016). If SPRs in speech and music reflect the use of a common limit cycle oscillator, then SPRs may be correlated across domains. Experiment 1 tests this prediction by comparing SPRs of pianists while they spoke and performed musical melodies.

A second framework emphasizes the peripheral role of *energy efficiency* based on the biomechanics associated with effector systems. This second framework predicts that spontaneous rates emerge based on biomechanical constraints, and similar rates are found for spontaneous rates using effector systems that abide by the same constraints. Results consistent with this view show that the stability of a rhythmic pattern varies with the biomechanical properties of movement (e.g., Goodman et al., 2000; Loehr and Palmer, 2007, 2009; Lopresti-Goodman et al., 2008; Nessler and Gilliland, 2009). For example, multi-finger tapping tasks indicate that index fingers generate more precise timing independently of other finger movements than do ring fingers, and coarticulation effects—in which one finger's movement trajectory is influenced by prior sequential finger movements—are larger for ring fingers and smaller for index fingers (Loehr and Palmer, 2007, 2009). In order to measure timing in rhythmic tapping independent of limb biomechanics and of perceptual feedback, the Spontaneous Motor Tempo (SMT) task was developed, in which a single (index) finger is used to tap a rhythm at a consistent rate on a hard surface (in the absence of any other perceptual feedback) under simple biomechanical conditions (using the most independent finger). Whereas, studies of the SMT task have ascribed individual differences in temporal precision to factors such as musical training or beat-deafness (Scheurich et al., 2018; Tranchant and Peretz, 2020), the wide range of individual differences in mean SMT rates remains unexplained; it was proposed that individuals' specific muscle movements are responsible for mean SMT differences across individuals (Fraisse, 1978). Several studies have reported SMT values that are more consistent within individuals than across individuals (Collyer et al., 1994; Dosseville et al., 2002). We test here whether individual differences in the SMT task are correlated with individual differences in the SPR task. We predict that inter-task correlations should be largest when similar limb movements are used: SMT rates should correlate more with pianists' music performance rates than with speech rates (a speech-based SMT task has not yet been proposed, presumably due to the task goals of reduced biomechanical constraints and absence of perceptual feedback). Experiment 1 tests this prediction by comparing SMT values from pianists' index-finger tapping with SPR values in the music and speech tasks.

A third framework predicts that production rates are governed by *communicative goals* associated with production. As noted earlier, conversational speech timing is oriented around reliable turn-taking (Wilson and Wilson, 2005). Short, uninterrupted utterances (with no hesitations or pauses) are optimal for this kind of behavior, so that pauses do not provide false cues for one's conversation partner that lead to a disruption of turn-taking. By contrast, Western forms of most music performance reflects more lengthy sequences (turns) and a slower overall pace than speech, based on a more regular beat, in order to permit synchrony of simultaneous productions with other performers; even in the case of less constrained solo performance, temporal regularity promotes entrainment and expectancy in listeners (Jones, 2018; Savage et al., 2021). As such, the communicative goals of music making are more often oriented around collective synchrony where pauses are pre-determined so that all voices maintain their synchrony. According to this view, SPRs in speech and music may not be correlated with each other due to differences in the communicative contexts typically associated with each domain, even though production rates in each domain may be internally consistent across repeated productions. Note that this third framework is not a null hypothesis, which would be the prediction that SPRs are inherently variable and do not lead to consistent rates even within a domain. This null hypothesis is unlikely for music performance, given high consistency of SPRs found in previous work (Zamm et al., 2016; Wright and Palmer, 2020), but it is possible in speech given current debates about whether regular rhythmic organization can account for speech timing.

## Experiment 1

Experiment 1 included monolingual English speakers from the University at Buffalo student community who had at least 6 years of private training on the piano. We measured participants' spontaneous production rates in three tasks: The production of sentences from memory, the production of melodies from memory (on a piano), and isochronous tapping (single-finger movements with no auditory feedback). Whereas, the music and speech production tasks included auditory feedback and measured SPRs, the tapping task included no auditory feedback and thus measured SMTs. Thus, comparisons across tasks provided an evaluation of whether associations in spontaneous rates are governed by effector systems (tapping and piano) or the use of auditory feedback (speech and piano).

## Method

### Participants

Nineteen participants from the Introductory Psychology research pool at the University at Buffalo participated in exchange for course credit. All participants were monolingual English speakers (using a standard American dialect) whose caregivers also spoke English as a primary language; participants had at least 6 years of private lessons on the piano, were in good vocal health during the session, and were able to sight-read (perform correctly without practice) a simple novel melody on the piano without errors. The mean age of participants was 19.05 years (*S* = 1.51, range = 18–25), and the mean years of private piano lessons was 9.08 years (*S* = 2.43, range = 6–14). Although participants were not fluent in any language other than English, all participants had some modest instruction in a different language (*M* = 6.16 years, *S* = 2.54, range = 2–13). Second language instruction was primarily in syllable-timed languages including Spanish (16 participants), French (4 participants), and Italian (1 participant). Two subjects also had instruction in Latin and one in German. All subjects reported having normal hearing and speech abilities.

### Stimulus Materials

Stimulus materials in Experiment 1 were drawn from previous studies of rhythm perception and production. Results from previous studies demonstrated that these items yield salient rhythms representative of each domain.

#### Speech Task

Twelve English sentences were used as experimental stimuli whose productions had previously been shown to exhibit salient and reliable stress properties in listeners who heard recorded utterances of the sentences (Lidji et al., 2011). Each sentence comprised 13 monosyllabic high-frequency words with stress patterns based on a trochaic metrical foot (i.e., binary strong/weak alternation). Sentences were presented on a computer monitor in the center of a PowerPoint slide that was positioned ~1 m in front of the participant. For a full list of sentences, see Appendix A.

#### Piano Task

Four isochronous novel melodies were chosen as stimulus materials for the music task; similar to the monosyllabic structure of the speech stimuli, all tones had the same duration (quarter notes) and their performances had previously been shown to generate reliable metrical stress patterns; two melodies were taken from Goebl and Palmer (2008) and the other two were drawn from Zamm et al. (2016). All melodies were 16 notes long, notated in a binary (4/4) meter (strong/weak alternation) for performance with the right hand (treble clef); the melodies varied in musical key (A minor, F Minor, G major, C major). Melodies were presented *via* standard music notation on a music stand positioned ~1 m in front of the participant. Notation for each melody can be found in Appendix B.

### Equipment

#### Speech Task

Participants were seated in front of an Acer S200HQL 20-inch LED computer monitor connected to a 3.6 gHz PC running Windows 10. The experiment was run using Matlab R2015a. Speech was recorded at a sampling rate of 44.1 kHz using a Shure WH30 head-mounted microphone connected through a Lexicon Omega I/O box.

#### Piano and Tapping Tasks

In the piano task participants performed on an electronic digital piano (Roland RD 700 SX). Sound was presented through Sennheiser HD 280 Pro headphones plugged into the digital piano. MIDI data from the digital piano were acquired *via* FTAP (Finney, 2001), a software program run on a Linux operating system. Auditory feedback during the piano task was based on the Grand Piano timbre setting. The tapping task used the same set-up, except the digital piano was muted so participants did not hear feedback when they pressed a piano key.

### Design and Procedure

Participants completed a screening task in which they memorized a 12-tone-long novel melody in the key of C major, presented in standard music notation. Participants had 3 min to practice the melody with the notation before it was removed and they were asked to perform the melody from memory. If participants performed the melody correctly from memory (without pitch errors) then the experiment continued, otherwise participants were excused and given credit for the amount of time that they participated. Participants were informed of this requirement at the beginning of the experiment.

Following the screening task, participants completed a music background survey. Next, participants completed one trial of the tapping task. For this task, participants were seated at the muted digital piano and were asked to tap on any key on the keyboard with the index finger of their dominant hand “at a regular and comfortable pace.” The experimenter waited ~40 s (using a hand-held timer) and then signaled the participant to stop tapping.

The speech and music tasks occurred next in two separate blocks, with the ordering of speech and music tasks counterbalanced across participants. The sentences were presented to each participant in one of two random orders, one order being the reverse of the other order. The order of the four melodies were counterbalanced across participants using a Latin square design.

In the speech task, participants were seated in front of a computer screen while wearing a headset microphone. Participants viewed each sentence on the computer screen until they had it memorized, at which point they pressed any key on the computer and the visual text was removed. Participants then produced the sentence three times from memory with no instructions concerning speaking rate. Sentences were not repeated as a continuous speech stream, but rather repetitions were delineated by a pause between the end and beginning of each sentence. For this reason, we considered each individual production as representing a single trial. There were thus three recorded trials for each sentence. Trial recordings for a sentence were repeated if participants experienced a memory lapse or made any speech errors. Participants repeated this sequence for each of the 12 stimulus sentences yielding 36 trials for the speaking task, each trial comprising a single repetition of one sentence. For each participant, therefore, we recorded a total of 468 syllables (13 syllables per sentence ×3 repetitions per sentence ×12 sentences).

In the piano task, participants were seated at the electronic digital piano in front of a computer screen and they put on headphones for auditory feedback. Participants viewed each melody in music notation and were allowed to practice the melody freely. During memorization, participants practiced the melody with their right hand, using the fingering indicated on the notation (1 = thumb, 2 = index finger, etc.). Participants informed the experimenter when they believed they had memorized the melody, at which point the experimenter removed the notation. Participants then played the melody four times from memory without pausing between each repetition. These four repetitions constituted a single trial. Participants then completed two more similar trials with the same melody, yielding three trials for each melody. This procedure was repeated for each of the four melodies, yielding 12 piano trials in total with each trial comprising four repetitions of a melody. For each participant, we recorded a total of 768 notes (16 notes in a melody ×4 repetitions of a melody per trial ×3 repetitions of each trial ×4 melodies).

Next, participants completed the isochronous tapping task again, using the same instructions as before. Following the tapping task, participants completed a language background survey. Participants were then debriefed and given course credit for the time that they participated in the experiment. The experiment took ~90 min to complete.

### Data Analysis

Spontaneous tempo was measured in music and speech production based on the mean inter-onset-intervals (IOIs) within each trial. For speech, syllable onsets were based on peaks in the amplitude contour that were associated with the perceptual onset of each syllable. The timing of these onsets was based on annotations made within Praat (Boersma and Weenink, 2013) and exported to text files. Twelve IOIs (13 onsets) per trial × 3 trials contributed to the mean speech measure per stimulus. For music, tone onsets were based on the timing of MIDI piano keypresses (16 tones × 4 repetitions = 64 per trial) measured by FTAP. We removed any repetition of speech or music sequences that contained errors, any IOI in between repetitions of a melody within a trial (sentences were not repeated within trials), and any IOI more than three standard deviations from the mean for that trial. The removal of these outliers led to discarding large pauses from estimates of speaking rate within trials, as is common in measures of articulation rate for speech. For tapping trials, we analyzed the first 40 IOIs (in the single trial) in the same way as for piano performance.

The primary analyses were correlations of SPRs within and across tasks. For each correlation, the parameter was participant (*N* = 19). Across-task correlations were based on mean SPRs across every trial for a participant and task. Within-task correlations were based on mean SPRs for a subset of trials within a task, as detailed in the Results section. Alpha corrections for comparisons across multiple correlations were carried out using the False Discovery Rate correction (FDR, Benjamini and Hochberg, 1995). Comparisons of mean SPR across tasks were carried out using repeated-measures Analysis of Variance (ANOVA), followed by Bonferroni-corrected pairwise comparisons. All statistical analyses were carried out in Rstudio version 1.2.1335 (R Studio Team, 2018), and all results reported as significant were significant following the relevant alpha correction.

## Results

**Figure 1:**
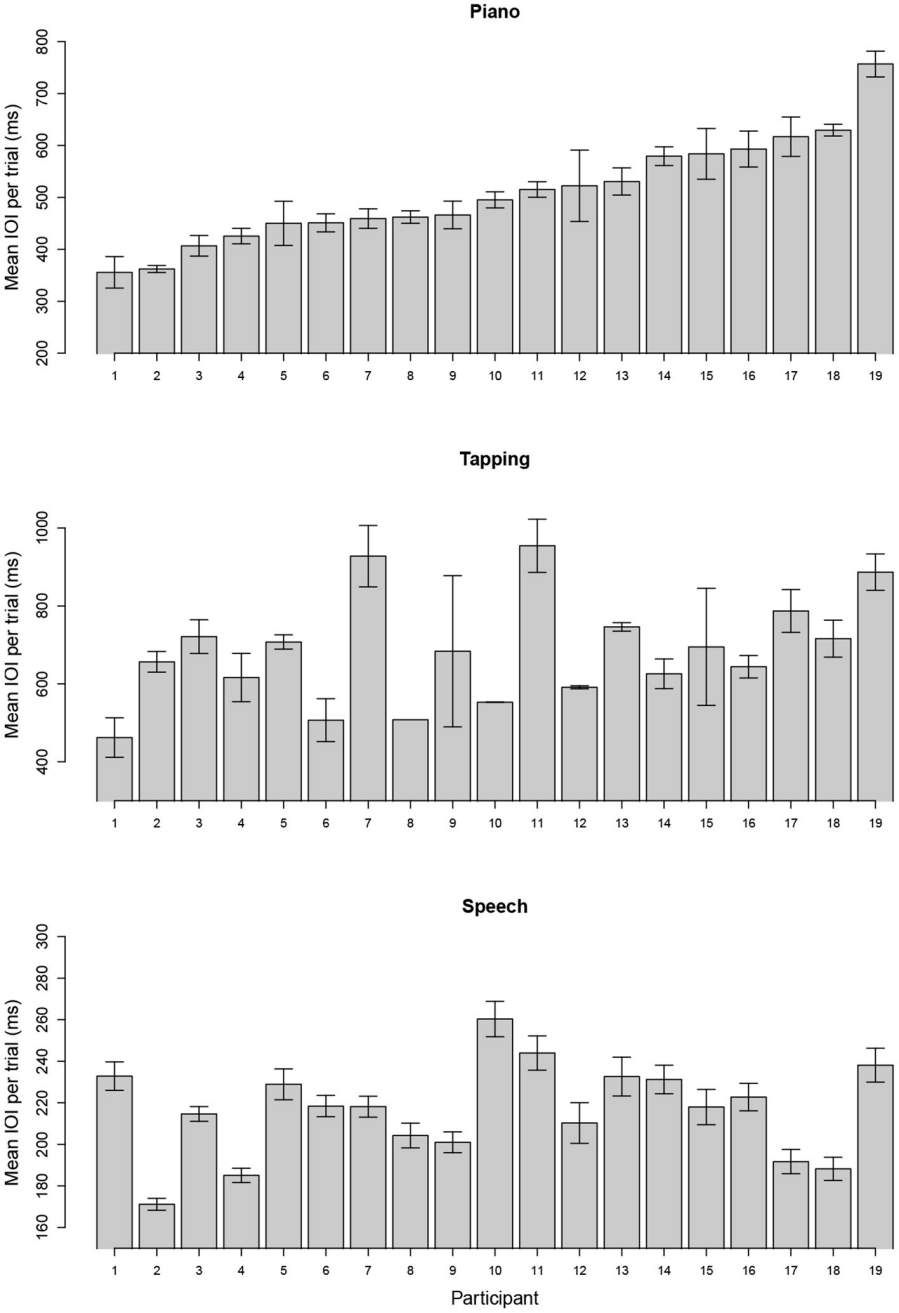
Mean (SE) of spontaneous rate across trials using mean interonset interval (IOI) for each individual when producing melodies (top), rhythmic tapping (middle), and speech (bottom). Units are expressed in milliseconds (ms), and participants are ordered in both graphs according to their spontaneous rate in music. Note that maximum and minimum Y-axis values vary across panels to show range of individual differences.

Spontaneous tempo was measured by the mean IOI produced for each individual, averaged across stimuli and trials. Figure 1 shows the distribution of mean rates across participants for each task; each bar represents the mean IOI across stimuli and trials for a single participant and task. Participants are ordered from fastest to slowest in each graph, based on the distribution of IOIs for the piano performance task. Production rates varied considerably across individuals in each task.

**Figure 2:**
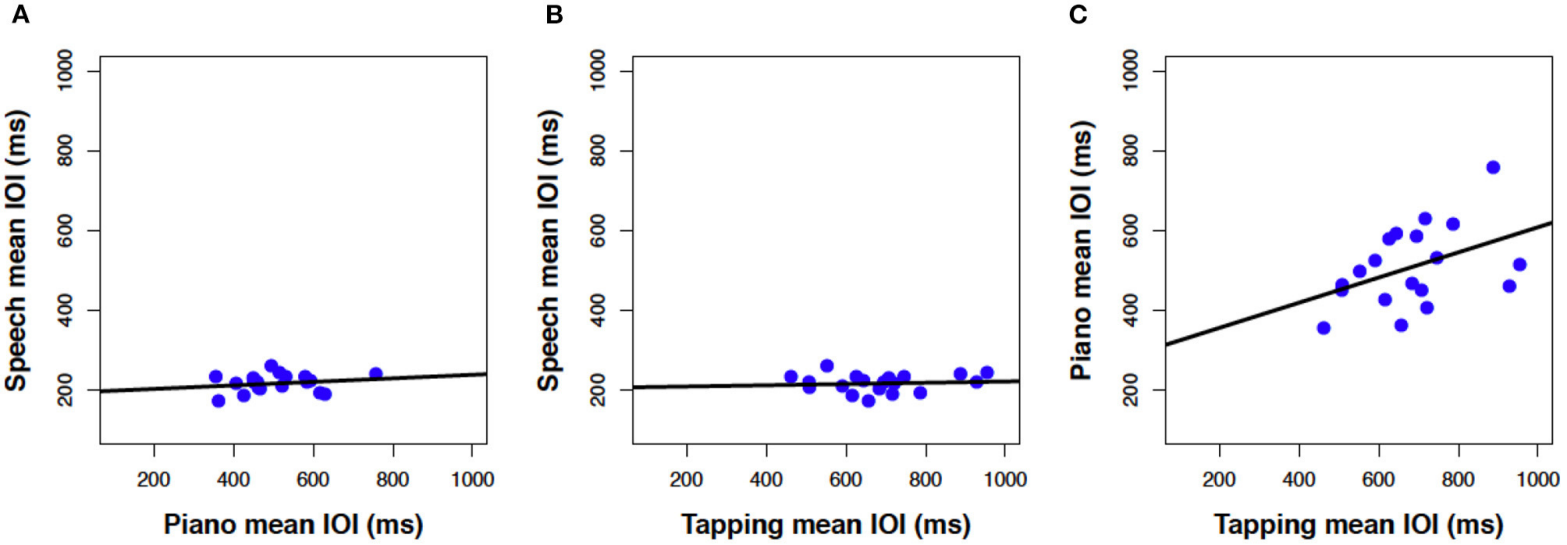
Scatterplots relating mean IOI (spontaneous rate) across piano and speech production **(A)**, rhythmic tapping and speech production **(B)**, and piano and rhythmic tapping production **(C)**. Lines reflect best-fitting linear regressions; each dot = one participant's mean IOI averaged across all trials for a given task (*N* = 19 in each panel).

Next, we consider how closely associated the spontaneous rates were across the three production tasks. Mean rates in Figure 1 are reproduced as scatterplots in Figure 2 across pairs of tasks (speech, piano, SMT) to illustrate these associations. Spontaneous speech rates did not exhibit a relationship with spontaneous piano rates, *r*_(17)_ = 0.20, *p* = 0.206, shown in Figure 2A, or with tapping rates, *r*_(17)_ = 0.10, *p* = 0.348, shown in Figure 2B. Slopes for best-fitting regression lines in each case were near zero (for piano vs. speech rate *B*_1_ = 0.04, SE = 0.05, for tapping vs. speech rate, *B*_1_= 0.02, SE = 0.04). On the other hand, spontaneous rates for piano performance and tapping (which shared effector movements) correlated significantly, *r*_(17)_ = 0.43, *p* = 0.033.^2^

**Figure 3:**
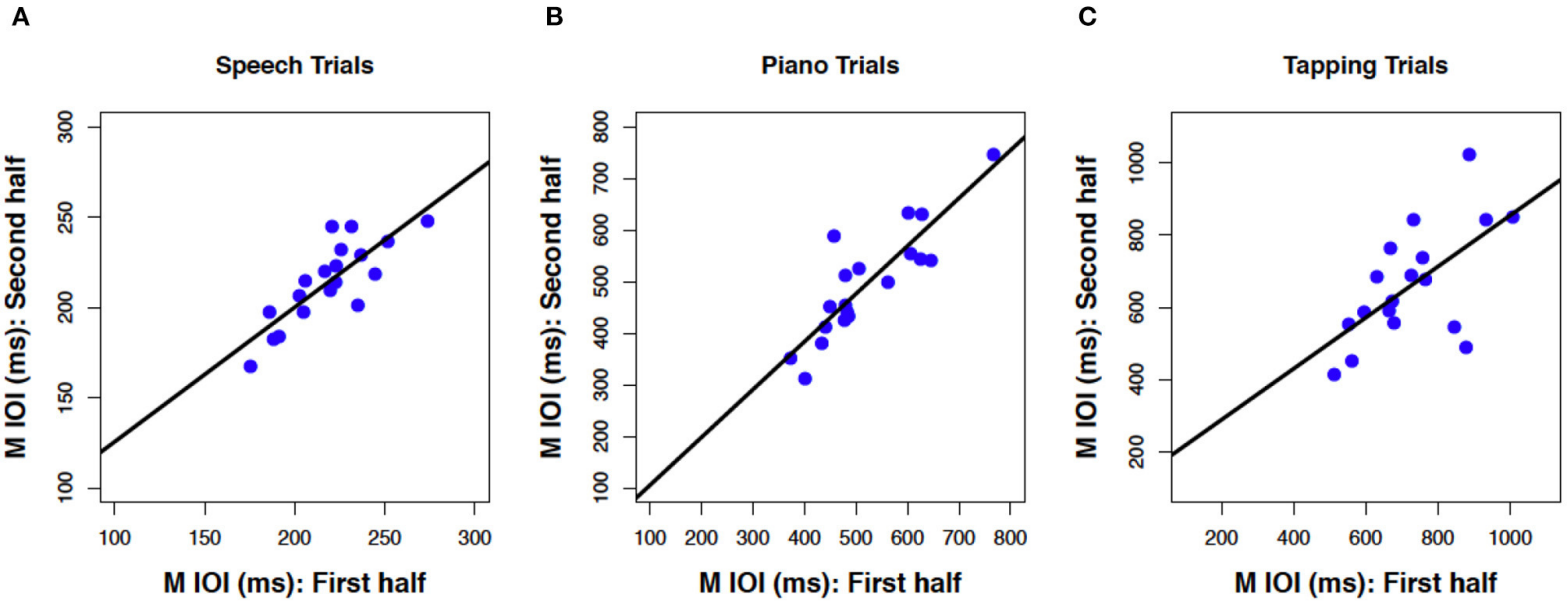
Scatterplots relating mean IOI across for the first vs. second half of the session in speech production **(A)**, piano production **(B)**, and rhythmic tapping **(C)**. Regression lines and parameterization as in Figure 2 (*N* = 19 **A,B**, *N* = 18 **C**).

Next, we consider whether spontaneous rates exhibit regularity within a given production task. Consistency of spontaneous speech rates across the experimental session was computed by averaging the mean IOI across speech trials in the first half of the session (sentences 1–6) and correlating that average with a similar measure based on speech trials in the second half of the session (sentences 7–12). The resulting correlation, shown in Figure 3A, was significant and positive, *r*_(17)_ = 0.81, *p* < 0.001. This is especially notable, given that different participants produced different sentences in the first and second half, based on the manipulated sentence orders. The best-fitting regression line comprised a slope of *B*_1_ =0.75 (SE = 0.13) and an intercept of *B*_0_ = 51 (SE = 29), indicating a modest amount of compression in individual differences from the first half to the second half of the session. Follow-up analyses confirmed that correlations between speech rates for individual items, based on the first and third repetition of each individual sentence, also reached significance (12 values, *r* ≥ 0.65 and *p* < 0.01 for each, see Appendix A for details).

Consistency of spontaneous rates within piano trials, shown in Figure 3B, was analyzed in the same way as for speech trials and also produced a positive and significant correlation, *r*_(17)_ = 0.87, *p* < 0.001. Again, this consistency is notable given the manipulated order of melodies across individuals from the first to second half of the sessions. The best-fitting regression line was close to unity, with a slope of *B*_1_ =0.93 (SE = 0.13) and an intercept of *B*_0_ = 13 (SE = 68). Consistency was also found within each melody across the first and third repeated trials (see Appendix B).

Finally, tapping trials measured at the beginning and the end of the experiment (Figure 3C), which constituted just two trials, exhibited a significant positive correlation across trials, albeit smaller in size than the other two associations, *r*_(16)_ = 0.62, *p* = 0.003. The reduced number of observations (*n* = 2 trials) and temporal separation between trials (beginning and end of session), along with potential interference from intervening speech and piano conditions, may have contributed to the smaller effect size found here than for consistency in piano and speech production (also, due to experimenter error, the data from one tapping trial for one participant was lost). The regression line included a slope of *B*_1_ = 0.71 (SE = 0.23) that indicated some compression, and an intercept that indicated slowing across trials (*B*_0_ = 148, SE = 167).

**Figure 4:**
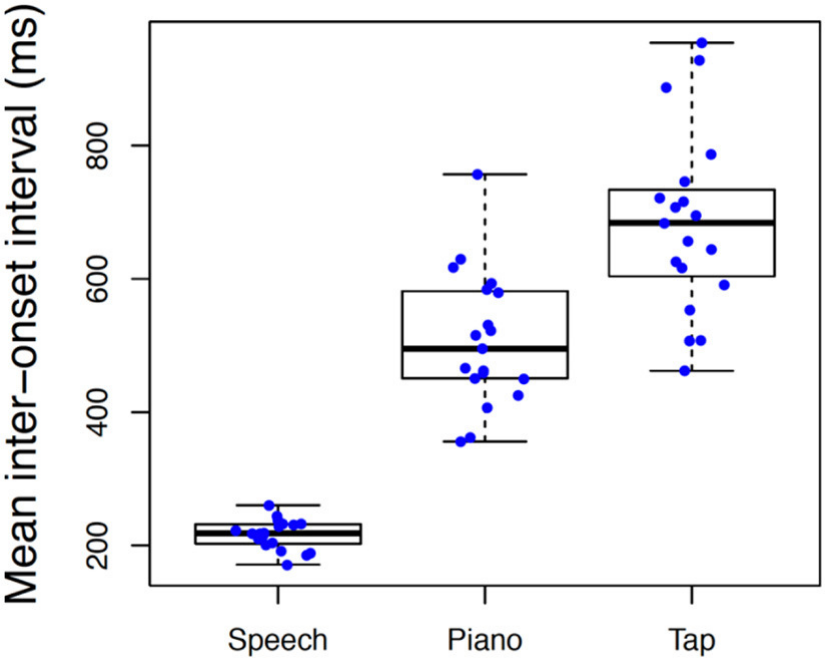
Boxplot illustrating differences in spontaneous rate across conditions. Individual boxplots represent the inter-quartile range (rectangle), median (dark horizontal line) and extreme scores (whiskers). Individual participant means are superimposed on box plots with random jitter along the x-axis to avoid occlusion of data points.

We also evaluated differences in spontaneous rate across tasks. These distributions are illustrated in Figure 4 using Box-Plots with individual data represented by each data point. Spontaneous rates varied significantly across tasks with a large effect size, *F*_(2, 36)_ = 141.88, *p* < 0.001, $\eta_{p}^{2}$ = 0.89, with slowest rates associated with tapping (*M* = 684, *S* = 137) intermediate for piano performance (*M* = 509, *S* = 101) and fastest for speech (*M* = 216, *S* = 22). All three pairwise comparisons were significant at *p* < 0.001. Variability of spontaneous rates across individuals was likewise more constricted in speech production than in piano production or tapping.

A potentially important factor in these analyses is the fact that the diversity of phonetic and syntactic structures in the 12 sentences may have led to lower levels of consistency among individual items than seen in the rhythmically and metrically regular musical melodies. To address this issue, we examined spontaneous rate correlations across each pair of sentences; results are shown in Table 1. One participant was missing data for two sentences; correlations involving these sentences were based on the 18 remaining participants. Although the magnitude of individual associations varied greatly, the majority of correlations were significant, with only 9 of 66 correlations (14%) failing to reach significance. Moreover, all non-significant correlations involved the same sentence (“Turn your head to look at me and tell me how you feel”). Follow-up analyses established that non-significant correlations between spontaneous rates in speaking and other tasks were not due to this single item. When the sentence “Turn your head” was excluded from the speech data, spontaneous speech rates still did not correlate significantly with spontaneous piano tempo, *r*_(16)_ = 0.31, *p* = 0.102, or with tapping rates, *r*_(16)_ = 0.12, *p* = 0.316. Correlations of M IOI across pairs of individual melodies for piano performance trials ranged from *r* = 0.66 to 0.87, with each correlation *p* < 0.001.

**Table 1:** Correlations of Mean IOI between each pair of sentences in Experiment 1.

	**Chips**	**Days**	**Eat**	**Girls**	**Jane**	**Kids**	**Night**	**Rats**	**Snacks**	**Tell**	**Turn**
Cats	0.52^*^	0.81^**^	0.59^**^	0.65^**^	0.83^**^	0.65^**^	0.42^*^	0.63^**^	0.71^**^	0.67^**^	0.15
Chips		0.69^**^	0.65^**^	0.79^**^	0.49^*^	0.67^**^	0.68^**^	0.74^**^	0.64^**^	0.70^**^	0.23
Days			0.77^**^	0.65^**^	0.91^**^	0.67^**^	0.71^**^	0.83^**^	0.83^**^	0.91^**^	0.23
Eat				0.63^**^	0.71^**^	0.66^**^	0.59^**^	0.56^**^	0.59^**^	0.72^**^	0.41^*^
Girls					0.51^*^	0.69^**^	0.58^**^	0.65^**^	0.75^**^	0.55^**^	0.39^*^
Jane						0.52^*^	0.66^**^	0.74^**^	0.76^**^	0.77^**^	0.37
Kids							0.61^**^	0.57^**^	0.62^**^	0.48^*^	0.23
Night								0.65^**^	0.61^**^	0.56^**^	0.19
Rats									0.80^**^	0.83^**^	0.37
Snacks										0.69^**^	0.27
Tell											0.35

*Sentences are identified by initial word (see appendix A). Correlations with “Jane” and “Tell” exclude one participant*.

* *p < 0.05*,

** *p < 0.01, after FDR correction*.

## Discussion

Experiment 1 provided evidence that participants produced a consistent spontaneous tempo within three different sequence production tasks: speech production, piano performance, and isochronous finger tapping (SMT task). Comparisons across tasks were based on shared/different auditory and motor features. Speech and piano production both involve auditory feedback, and constitute SPR tasks, whereas tapping does not. By contrast, piano and tapping tasks share a common effector system, using finger and hand movements, whereas speech uses vocal articulators.

Overall, results from Experiment 1 supported the idea that spontaneous rates reflect biophysical properties of effector systems. Production rates in piano performances correlated significantly with tapping rates, but neither of these tasks correlated with speaking rates. Significantly, individuals exhibited internal consistency within all three tasks. Spontaneous speaking rates correlated across the first and second half of the session, and even across most of the individual sentences. Thus, these findings do not suggest the absence of reliable rhythmic organization in speech. Instead, the findings suggest that speech timing may be governed by factors that are distinct from piano or tapping, consistent with the theoretical proposal that communicative goals guide temporal organization.

A potential limitation of Experiment 1 is the use of complex sentences that were less rhythmically consistent than the music or tapping stimuli. Although the sentences used in Experiment 1 were designed to be rhythmic, they also presented non-trivial memory demands, which may have prevented the emergence of salient rhythmic properties. Therefore, in Experiment 2 we simplified the speech production task in order to maximize its rhythmic properties.

## Experiment 2

Experiment 2 focused on spontaneous tempo in piano and speech production tasks. Whereas, the piano stimuli in Experiment 2 were nearly identical to Experiment 1, the speech task was changed considerably to enhance similarity across these tasks and to elicit the maximum rhythmicity in speech while still retaining critical features that distinguish speech from music. First, we had participants produce shorter phrases, rather than the long sentences of Experiment 1, in order to reduce memory load that may have occluded potentially rhythmic speech patterns during production. Second, we had participants produce these phrases many times in a cyclical fashion (Cummins and Port, 1998) and we measured spontaneous rate by aggregating across these repetitions. Recent research suggests that the repetition of speech causes listeners to perceive more song-like qualities (Deutsch et al., 2011; Tierney et al., 2013, 2018; Falk et al., 2014; Vanden Bosch der Nederlanden et al., 2015). We hypothesized that repeated productions may lead to SPRs more similar to those in music performance tasks. Finally, we selected a new set of stimuli from a database of samples previously found to yield perceptual transformations from speech to song (Tierney et al., 2013).

The changes in design for Experiment 2 addressed a potential concern in Experiment 1 that the number of repetitions for each sequence differed across speech and music production. Whereas, participants in Experiment 1 produced each of 12 sentences only once per trial with three trials per sentence, those participants produced each of four piano melodies four times per trial with three trials per melody. Piano performance thus included more pattern repetition which may have stabilized timing and led to more reliable estimates of spontaneous rate. In Experiment 2, pattern repetition was better equated across piano and speech production.

## Method

### Participants

Experiment 2 was conducted at McGill University in Montréal. Nine adult bilingual pianists were recruited for this study. Participants' ages ranged from 19 to 37 (*M* = 22.33) and all were right-handed except for one. Linguistic criteria for inclusion were knowledge of English and French, with one of those as the first language learned (L1), no additional languages learned at the same time, and no acquisition of languages using Linguistic tones or Mora timing. Eight of nine participants were English-L1 and one was French-L1. One of the English-L1 participants considered themselves less fluent in French. Three of the English-F1 speakers reported some knowledge of additional languages that included Italian, Spanish, and Hebrew. Musical criteria for inclusion were at least 6 years of private piano instruction, and no history of neurological or hearing conditions. The amount of private piano lessons in years ranged from 8 to 15 (*M* = 11.56). All participants completed audiometric screening tests administered at the beginning of the experiment that showed hearing thresholds of <30 dB SPL for the 125–750 Hz range of frequencies in the musical melodies.

### Stimulus Materials

#### Bilingual Dominance Scale

All participants completed the 12-item Bilingual Dominance Scale (Dunn and Fox Tree, 2009), to assess their relative usage of English and French. The scale ranges from −30 (French-dominant) to +30 (English-dominant) with 0 indicating equal dominance of the two languages. The ratings for the English-L1 participants ranged from 10 to 22 (M = 16.7) and the rating for the French-L1 participant was −19. All participants learned the L2 language by age 10 and all had some years of schooling in both English and French.

#### Speech Task

Four speech phrases were derived from rhythmic speech stimuli that were shown to generate the speech-to-song illusion in Tierney et al. (2013). The speech-to-song illusion occurs when repetitions of a spoken phrase cause that phrase to sound as if it were sung (Deutsch et al., 2011); such phrases have rhythmic properties that should allow easy repeated production. We modified the original phrases to further enhance ease of pronunciation by our target population. Each phrase consisted of eight syllables, with position of stress varying in location and in pattern (which could be binary or ternary). The two binary phrases included: “Cakes are good until tomorrow,” “To choose between the final two,” and the two ternary phrases included: “To convince her to change her plans,” and “The queen continued to struggle.” Further details can be seen in Appendix C.

#### Piano Task

The four melodies from Experiment 1 were used in Experiment 2. Two melodies were modified slightly, as shown in Appendix D. The fingering in measure 2 of melody 2 was altered to promote more fluent production, and the key of melody 4 was changed to enhance the diversity of the melodies' frequency range. Further details can be seen in Appendix D.

### Equipment

#### Audiometric Screening

Participants performed a hearing screening with a Maico MA40 audiometer.

#### Speech Task

Sentences were presented on a Dell 2408WPFb Monitor and utterances were recorded at 44.1 kHz by a Shure Beta 54 head-mounted microphone and sent to a MOTU 828 MKII audio interface and recorded to computer.

#### Piano Task

Participants performed melodies on a Roland RD-700NX keyboard, with keystrokes recorded *via* MIDI signals by FTAP (Finney, 2001) on a Dell Precision T3600 PC running Linux (Fedora 18; Raleigh, NC). Participants listened to live feedback of their performances through AKG K71 headphones, and a tone generator (Roland SD-50) using a piano timbre (GM2 sound bank) was used to produce the sounds from the key presses on the keyboard.

### Design and Procedure

During each testing session, participants were briefed with consent forms that outlined the experimental procedure. Participants then completed screening tasks to determine that they met eligibility requirements. First participants underwent an audiometry screening test. Next, participants completed a piano sight-reading memory task similar to Experiment 1, to ensure their ability to memorize from music notation.

Following successful completion of the initial hearing screening and memory tests, participants completed a speech task and a music task, with the ordering of these tasks counterbalanced across participants. Both the order of sentences and the order of melodies were counterbalanced across participants according to a Latin Square.

Speech production trials began by presenting participants with a phrase on a computer screen which they were asked to memorize. When they indicated that they had memorized it, the phrase disappeared and the participant was asked to produce it from memory. Following word-perfect memorization, participants repeated the phrase eight times without stopping to complete a single trial. Participants were instructed to speak at a comfortable and consistent rate of production, to speak into the microphone, and to repeat the phrase as if they were speaking to someone who didn't understand what was said, or as if they were speaking to an automatic voice recognition program. The experimenter gave verbal instructions to begin and end each trial so that participants did not have to keep track of the number of repetitions. Following a practice trial, participants completed three successive trials for each phrase. Thus, there were 12 recorded speech trials per participant (3 per phrase), with each trial comprising eight repetitions of the phrase. For each participant, we recorded a total of 768 syllables (8 syllables per phrase ×8 repetitions of a phrase per trial ×3 repetitions of each trial ×4 phrases).

Piano performance trials were structured similarly to Experiment 1, and to the speech trials of Experiment 2. Upon presentation of a melody in music notation, participants were given time to practice the melody in order to commit it to memory. Following pitch-perfect accuracy of performance without the notation, participants began the trials. Each trial consisted of four repetitions of each melody performed without pausing between repetitions. During each trial, participants were instructed to perform at a comfortable and consistent rate and to repeat the melodies without pauses between repetitions. When the participant had performed the four repetitions, the computer stopped producing audio feedback, indicating that the trial was over. Three successive trials of four repetitions each were performed for each melody. As in the speech task, we recorded 12 piano performance trials per participant. For each participant, we recorded a total of 768 notes (16 notes in a melody ×4 repetitions of a melody per trial ×3 repetitions of each trial ×4 melodies), the same number as in Experiment 1.

Between speech and music tasks, participants completed questionnaires about their musical background and bilingual dominance (Dunn and Fox Tree, 2009). Questions regarding musical background included number of years playing piano, number of years of private instruction on the piano, number of hours per week currently playing piano, description of ensemble work, and a self-rating of sight-reading ability. Questions of bilingual dominance included which language the participant first learned, age at which the participant first felt comfortable speaking English and French, the language predominantly spoke at home, number of years of schooling in French and English, and loss of fluency in any language.

Following completion of the speech and music tasks and the questionnaires, the participants were debriefed on the details regarding the experiment and were given the opportunity to ask questions. Participants received a nominal fee for their participation in the study. The entire experiment lasted ~1 h.

### Data Analysis

Data in Experiment 2 were analyzed in the same way as in Experiment 1. Unlike Experiment 1, spoken phrases were repeated multiple times in a trial. Therefore, we removed all IOIs that elapsed between repetitions of a phrase.

## Results

**Figure 5:**
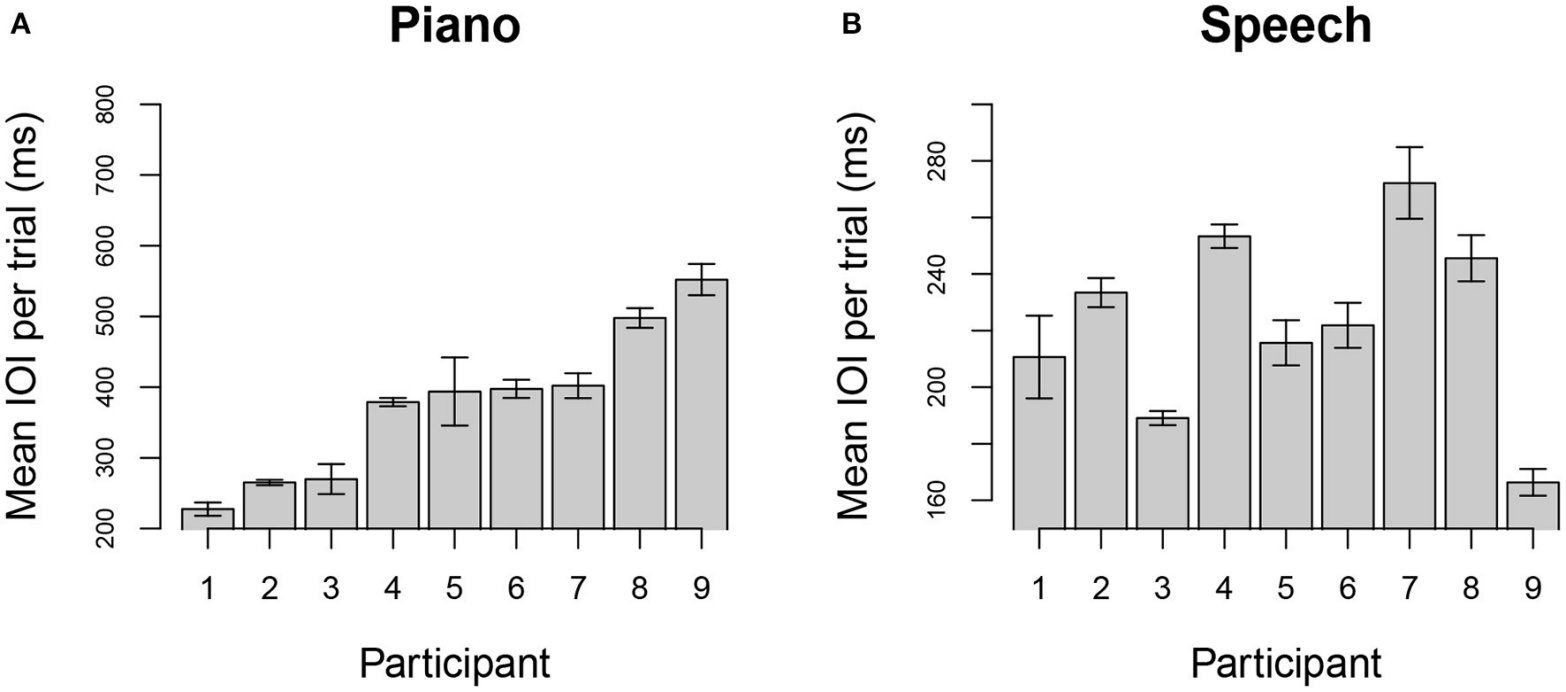
Mean IOI (SE) of spontaneous rates by individual for melodies **(A)** and speech **(B)**. Units are expressed in milliseconds, and participants are ordered in both graphs from fastest to slowest spontaneous rates in the music task. Note that y-axis limits vary to show range of individual differences. Participant #8 was the French L1 participant.

We first assessed the range of spontaneous rates (measured by mean IOI) within speech and piano tasks. Figure 5A shows the mean IOI ordered across participants from fastest (left) to slowest (right) music IOI. Figure 5B shows the speech rates for the participants, ordered the same as for the music rates.

**Figure 6:**
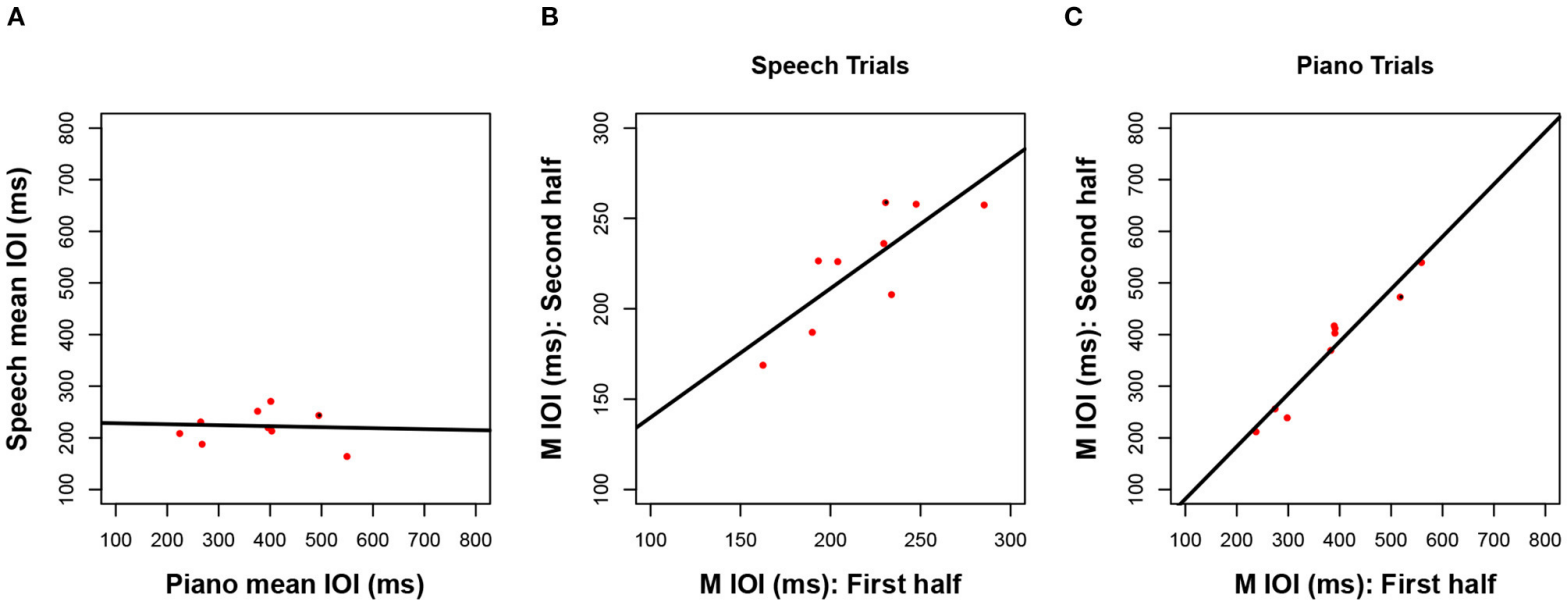
Scatterplots of mean IOI across speech and piano tasks **(A)**, within speech **(B)** and within piano **(C)** for bilingual speakers (*N* = 9 each panel). The darkened data point highlights the French L1 participant.

Next we evaluated the association between spontaneous rates of speech and music, shown as a scatterplot in Figure 6A. As in Experiment 1, the resulting correlation was not significant, *r*_(7)_ = −0.06, *p* = 0.562, with a slope near zero *B*_1_ = −0.02 (SE = 0.11).

Consistency of spontaneous speech rates across the experimental session was computed by averaging the mean IOI across speech trials in the first six trials (phrases 1–2) and correlating that average with a similar measure based on speech trials in the second six trials (phrases 3–4). No differences as a function of binary vs. ternary metrical organization in speech trials was apparent, and so we averaged across this factor for all analyses reported here. The resulting correlation, shown in Figure 6B, was significant and positive, *r*_(7)_ = 0.81, *p* = 0.004. The best-fitting regression line comprised a slope of β_1_= 0.72 (SE = 0.20) indicating a modest amount of compression in individual differences from the first half to the second half. Follow-up analyses confirmed that consistency in spontaneous rates also held at the level of individual items, using correlations of spontaneous rates between the first and third trials for each individual phrase (phrase 1 *r* = 0.89, phrase 2 *r* = 0.97, phrase 3 *r* = 0.88, phrase 4 *r* = 0.93, each *p* < 0.01).

Consistency of spontaneous rate was computed across piano trials 1–6 and trials 7–12 (Figure 6C) and likewise yielded a significant positive correlation, *r*_(7)_ = 0.96, *p* < 0.001, with a slope at unity *B*_1_ = 1.02 (SE = 0.11). This correlation held at the level of individual items. It is worth noting that the single French-dominant participant, highlighted by a darkened center circle in Figure 6, was not an outlier for either of these within-task correlations. Similar to speech, correlations across the first and third trials for individual melodies were significant (melody 1 *r* = 0.92, melody 2 *r* = 0.98, melody 3 *r* = 0.96, melody 4 *r* = 0.88, each *p* < 0.01).

**Figure 7:**
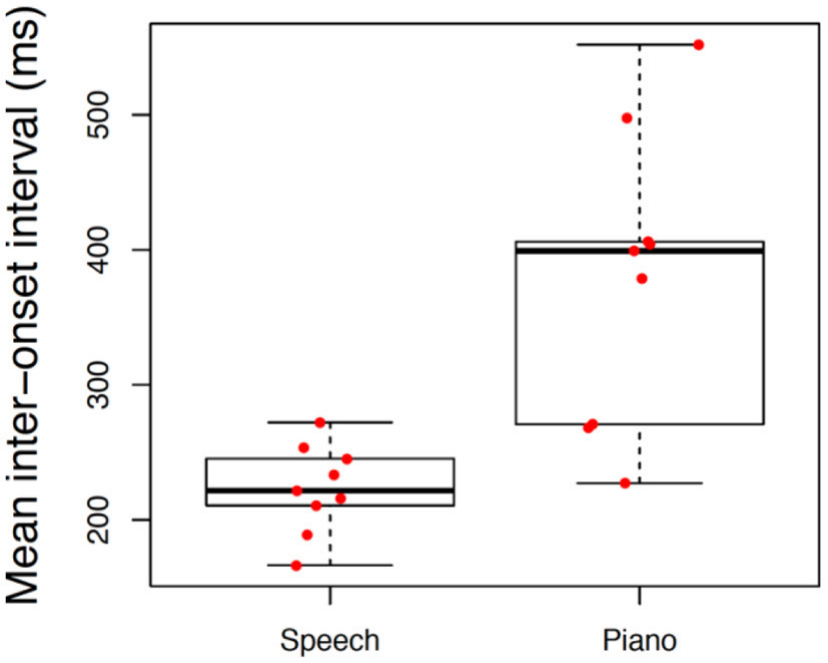
Boxplot illustrating differences in spontaneous rate across conditions. Individual boxplots represent the inter-quartile range (rectangle), median (dark horizontal line), and extreme scores (whiskers). Individual participant means are superimposed on box plots with random jitter along the x-axis to avoid occlusion of data points.

We next compared the mean IOIs across tasks. Similar to Experiment 1, mean IOIs for speech were significantly faster (*M* = 223, *S* = 33) than mean IOIs for piano (*M* = 378, *S* = 108), *t*_(8)_ = −4.04, *p* = 0.004 (Student's t), as shown in Figure 7. Like Experiment 1, variability of spontaneous rates across individuals was more constricted in speech production than in piano production.

One motivation for Experiment 2 arose from differences in the consistency of SPRs across the complex speech items used in Experiment 1 (see Table 1), which may have added noise to the measure of spontaneous tempo. We conducted a similar analysis of correlations across individual phrases within participant for Experiment 2. Results are shown in Table 2. As can be seen, every individual item was produced at a rate that correlated significantly with the rate of every other item. The intercorrelations among stimuli based on a similar stress pattern (binary or ternary) were higher (*M* = 0.865) than among stimuli with different stress patterns (*M* = 0.745), although this difference was not significant (*p* = 0.54, test of independent *r*'s). Correlations of M IOI across pairs of individual melodies for piano performance trials ranged from *r* = 0.77 to 0.95, with each correlation *p* < 0.001. In sum, Experiment 2 replicated the results of Experiment 1 with simpler rhythmic speech stimuli.

**Table 2:** Correlations of Mean IOI between each pair of sentences in Experiment 2.

	**Choose (B)**	**Convince (T)**	**Queen (T)**
Cakes (B)	0.88^**^	0.91^**^	0.82^**^
Choose (B)		0.93^**^	0.65^*^
Convince (T)			0.84^**^

*Sentences are identified by first content word. B = binary metrical organization, T = ternary metrical organization*.

* *p < 0.05*,

** *p < 0.01 after FDR correction*.

Associations of mean IOI across different spoken utterances may reflect the use of a stable timekeeping mechanism; or they could reflect a central tendency for the timing of individual events that are themselves produced with inconsistent (e.g., randomly varying) timing across utterances. We therefore analyzed the contrastive stress timing of syllables using the normalized pairwise variability index, or nPVI (Grabe and Low, 2002), which measures the mean absolute difference between pairs of IOIs, standardized by the overall time interval formed by each pair. Because each phrase in Experiment 2 had a distinct metrical structure, we focused on correlations within each sentence that compared mean nPVI across the first and third trials for a given phrase. These correlations were positive and significant at *p* < 0.01 for each phrase (phrase 1 *r* = 0.93, phrase 2 *r* = 0.95, phrase 3 *r* = 0.83, phrase 4 *r* = 0.98). The consistency in the amount of contrastive stress used across different productions of a phrase provides further evidence that speech timing in Experiment 2 was based on a stable rhythmic organization.

### Results for Combined Experiments

**Figure 8:**
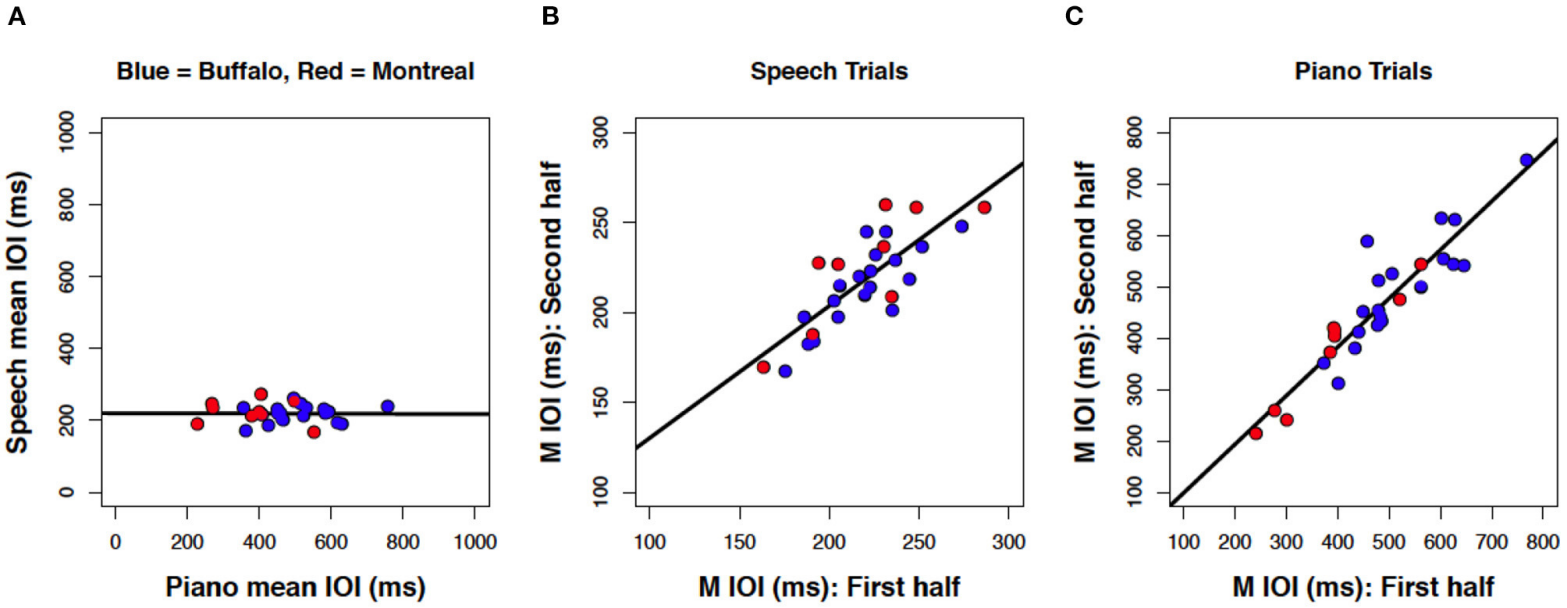
Scatterplots of mean IOI between speech and music **(A)**; within speech **(B)** and within piano **(C)**, including data from both Experiment 1 (blue) and Experiment 2 (red). Each dot represents one participant (*N* = 28 each panel).

Finally, we aggregated data sets across Experiments to address how consistent the results were from two speaker samples (monolingual/bilingual) tested with different speech stimuli (complex/simple). We focused on correlations within and across domains for mean IOIs in speech and piano production. As shown in Figure 8, the results across experiments were highly consistent, and both data sets fall on a common slope. Correlation of SPRs across domains (Figure 8A) yielded a regression line that was virtually flat, *r*_(26)_ = 0.01, *p* = 0.478. By contrast, within-task correlations yielded significant positive correlations for speech production, *r*_(26)_ = 0.80, *p* < 0.001 (Figure 8B), and piano performance, *r*_(26)_ = 0.92, *p* < 0.001 (Figure 8C).

We also analyzed the results shown in Figure 8 using the Bayes Factor criterion (Rouder et al., 2009), an estimate of how much support a result provides for both the null and the alternative hypotheses. Sample sizes in the present studies were relatively small due to the difficulty of recruiting participants matching the substantial criteria for inclusion (with restrictions based on both musical experience and language background). Bayesian statistics do not make the same assumptions as frequentist statistics about sample size and thus offer a useful validation check (Kruschke, 2010). Using the correlation BF function from the BayesFactor package in R Studio (Morey et al., 2009), the Bayes factor criterion for the likelihood of the alternative hypothesis (positive linear correlation) over the null (no correlation) was over 1,000 for within-task correlations (Figures 8B,C), whereas the likelihood of the null over the alternative hypothesis approached zero. Thus, the Bayes Factor results indicated considerable support for the positive linear correlations within speech and music rates. By contrast, the Bayes factor for the likelihood of the alternative hypothesis over the null was BF = 0.225 for the correlation across speech and music (Figure 8A), with the inverse BF = 4.41. We therefore found modest support for the lack of correlation in spontaneous rates across tasks (Rouder et al., 2009).

## Discussion

Experiment 2 replicated the primary findings from Experiment 1 with bilingual speakers and shorter sentences: strong within-participant consistency in spontaneous rates for speech and music, and no association in spontaneous rates across domains. As in Experiment 1, syllables were produced at a faster rate than were successive notes in piano performances. The rhythmically consistent English speech stimuli used in Experiment 2 yielded greater consistency in bilingual English/French speakers' SPRs than was found in Experiment 1, yet this increased regularity was limited to associations across speech trials and did not affect associations across speech and music production.

## General Discussion

Musically trained individuals—both monolingual (Experiment 1) and bilingual (Experiment 2) English speakers—exhibited consistent SPRs within the domains of speech and music. Each individual's spontaneous speech rate was found to be consistent across utterances, and their spontaneous musical tempo was consistent across melodies. However, the SPRs did not correlate across domains. We evaluate these results in light of the theoretical frameworks described in the introduction.

One theoretical perspective proposed that spontaneous rates originate from an endogenous rhythm, based on the stable state of a central limit cycle oscillator (McAuley, 1995; Large and Jones, 1999; Large and Palmer, 2002; Jones, 2018). The consistency of SPRs within each domain is consistent with this view; however, the lack of correlation across speech and music domains suggests that those SPRs cannot be accounted for by a single centrally controlled limit-cycle oscillator. It is possible that SPRs for music and speech reflect stable temporal states, but are not based on a single common referent. Nevertheless, it is important to underscore the theoretical importance of our finding that both speech and music SPRs are highly consistent within individuals. Experiment 2 further demonstrated that speakers consistently vary patterns of stress within an utterance. Speech timing does seem to reflect the use of a consistent rhythmic framework, as in music, although the stable state for speech rhythms appears to be independent of that for music.

The fact that piano SPRs correlated significantly with SMTs from isochronous tapping and not with speech SPRs converges with the theoretical perspective that spontaneous rates reflect biomechanical constraints within an effector system (Bernstein, 1967; von Holst, 1973; Haken et al., 1985; Kelso, 1995). Limb-specific anatomical and biomechanical constraints influence timing; for example, the timing of leg movements in walking is thought to be determined by natural motor resonances of specific limbs, which are related to physical characteristics such as limb length and weight (Goodman et al., 2000; Nessler and Gilliland, 2009). Pianists' finger and hand movements are similarly influenced by interdependences in shared muscles and tendons that affect timing of sequential movements in ways similar to coarticulation effects in speech (Loehr and Palmer, 2009; Goebl and Palmer, 2013). The fact that tapping rates were significantly slower than piano SPRs, yet significantly correlated, is consistent with findings that repetitive single-finger actions of the SMT task do not permit coarticulation (preparatory finger movements for the next element) as do SPR tasks. Consistent with this, Scheurich et al. (2018) found correlations between the size of pianists' wrist ulna (itself correlated with the physical frame size of a participant) and participants' single-finger tapping rates, but not with multi-finger piano performance rates.

The dissociation of reliable differences in piano and speech SPRs is consistent with the framework that SPRs reflect the communicative goals associated with a given domain. In particular, the slower rates of music SPRs than speech SPRs may reflect the demands of synchronicity in the music domain that require significant motor planning in order to synchronize tone onsets, a commonality that is rarely found in the speech domain. Consistent with this claim, we observed high consistency in preferred production rates in the piano task, and average spontaneous rates across participants displayed a large range of tempi. By contrast, speech SPRs, although just as consistent, did not display as wide a range of individual differences. This may be related to the need to engage in rapid and efficient speech that is unconstrained by simultaneous synchronization processes.

It is possible to reconcile these theoretical accounts of production rate differences in speech and music. The biomechanical constraints of different effector systems may reflect different endogenous oscillators with different natural frequencies. For example, speech tempo measures may indicate that the vocal apparatus operates with a high (fast) resonance or natural frequency (van Lieshout, 2017), relative to the effector systems used for music performance and index-finger tapping. In this view, endogenous rhythms are still at play, but instead of a single limit-cycle oscillator, multiple coupled oscillators with different resonance frequencies are associated with different effector systems. For example, Peper et al. (2004) proposed a bidirectional coupling between a limit cycle oscillator at the neural level and oscillators at the effector level, that accounted for resonance frequency differences in interlimb coordination. Several investigations of multi-limb movements have employed mathematical models of coupled oscillators, with each oscillator reflecting natural frequencies associated with different effector movements (Kelso and Jeka, 1992; Fuchs et al., 1996). The unique communicative goals of speech and music behaviors may be served by different resonance frequencies and couplings among the different effector systems.

Further investigation of the role of effectors would come from an analysis of SPRs in singing vs. speaking, which we plan to pursue in future research. The present study compared speaking and piano performance as an initial step for several reasons. First, we wanted to compare tasks representative of music and spoken language whose rhythmic properties have been validated in previous studies. That was the case for stimuli used here. Second, we wanted participants to generate patterns based on visual prompts that were not associated with prior auditory examples. Sight reading on the piano is a well-practiced task for individuals with piano training, whereas singing from notation can be very difficult, even for individuals with formal singing training. Although singing a familiar song from memory offers a method of singing that is free of sight reading demands, this type of task can be associated with specific heard examples and tempos which would influence participants' chosen production rates. Finally, we wanted to select participants with formal training to avoid measuring disfluencies. It is easier to find participants with formal training on the piano than formal training in singing (which is usually learned implicitly).

One limitation of the present research was in the use of scripted speech for measuring speech SPRs, which may not reflect SPRs for extemporaneous speech. However, there are advantages of studying rates of memorized speech over extemporaneous speech. First, extemporaneous speech typically involves bouts of production that alternate with pauses used to plan future utterances. These pauses reflect memory retrieval and planning rather than temporal organization of production, and thus may not directly measure an endogenous rhythm. Additionally, extemporaneous speech often contains disfluencies or changes in planned utterances that disrupt timing and are difficult to identify as errors or intentions. Scripted speech avoids such problematic epochs in a data set. Finally, due to controlled phonetic and syntactic structures, scripted speech avoids the possibility that these sources of variability may contribute to SPRs for different participants (Jacewicz et al., 2009).

In conclusion, two experiments measured spontaneous production rates with different speaker populations and different speech stimuli, and produced strongly converging results. First, both monolingual and bilingual individuals exhibited highly consistent SPRs both while speaking and while performing the piano, showing some evidence for endogenous rhythms that transcend specific sequences and time (experimental duration). Second, SPRs in speech and music were independent of each other. Finally, spontaneous rates of tapping and of music performance (both based on finger movements) were correlated. Timing of speech and music may reflect different uses of effector systems and communicative goals, leading to distinct SPRs, while productions within both domains exhibits a consistent rhythmic basis.

## Data Availability Statement

The raw data supporting the conclusions of this article will be made available by the authors, without undue reservation.

## Ethics Statement

The studies involving human participants were reviewed and approved by University at Buffalo, SUNY, and McGill University. The patients/participants provided their written informed consent to participate in this study.

## Author Contributions

PP wrote manuscript, designed Experiment 2, and data analysis. EG wrote first draft of Experiment 1, designed Experiment 1, data collection and analysis for Experiment 1, and manuscript revisions. AF wrote first draft of Experiment 2, collected data for Experiment 2, data analysis for Experiment 2, and manuscript revisions. CP designed both experiments, data analysis, and manuscript revisions. All authors contributed to the article and approved the submitted version.

## Conflict of Interest

The authors declare that the research was conducted in the absence of any commercial or financial relationships that could be construed as a potential conflict of interest.

## Footnotes

^1^In most frameworks, syllables are considered to be the rhythmic unit underlying non-Mora timed languages (but see Cummins, 2012b; e.g., Peelle and Davis, 2012; Poeppel and Assaneo, 2020), and thus roughly analogous to notes and chords in music. At the same time, temporal organization is clearly complex and multi-leveled in each domain.

^2^The two tapping trials occurred at the beginning and end of the session. Whereas the correlation of spontaneous rates from piano correlated significantly with the tapping trial at the end of the study, *r*_(16)_ = 0.41, *p* = 0.047, the correlation with the tapping trial at the beginning of the session fell short of significance, *r*_(16)_ = 0.34, *p* = 0.081.
