# Supplementary material for: Spontaneous Production Rates in Music and Speech
Source: Front Psychol. 2021 May 31;12:611867. doi: 10.3389/fpsyg.2021.611867 (PMC8200629; doi:10.3389/fpsyg.2021.611867)
Supplement: Supplementary file 1 [file Data_Sheet_1.docx]

Appendix A

**Sentence Stimuli in Experiment 1**

| Sentence | M | R-1v3 |
| --- | --- | --- |
| Cats and dogs are pets of course but treat them as your friends. | 213.16 | 0.74 |
| Chips and cake are good as treats, but some will make you sick. | 219.06 | 0.47* |
| Days are bright with lots of light, but nights are cold and dark. | 227.46 | 0.83 |
| Eat some bread, said Mom to Jeff, so you grow big and tall. | 236.42 | 0.62 |
| Girls and boys who trip and fall can hurt their hands and knees. | 214.81 | 0.81 |
| Jane and Tom were pals as kids, but then they fell in love. | 222.37 | 0.70 |
| Kids who play in mud and dirt will have to wash their pants. | 199.02 | 0.63 |
| Night in spring is much too cool to wear a dress that short. | 224.54 | 0.71 |
| Rats and mice are in the grass, but some run through the house. | 213.05 | 0.76 |
| Snacks are good to bring to school but too much food is bad. | 216.65 | 0.62 |
| Tell the girls to keep their poise when strange young men are near. | 237.44 | 0.78 |
| Turn your head to look at me and tell me how you feel. | 177.26 | 0.80 |
|  |  |  |

* One participant missing data for this correlation, *p* < .05 for this correlation, *p* < .01 for all others.

*Note:* M = mean spontaneous rate (ms) across trials and participants, R-1v3 = correlation of M IOI between the first and third repetitions of a sentence across participants.

Appendix B

**Melody Stimuli in Experiment 1**


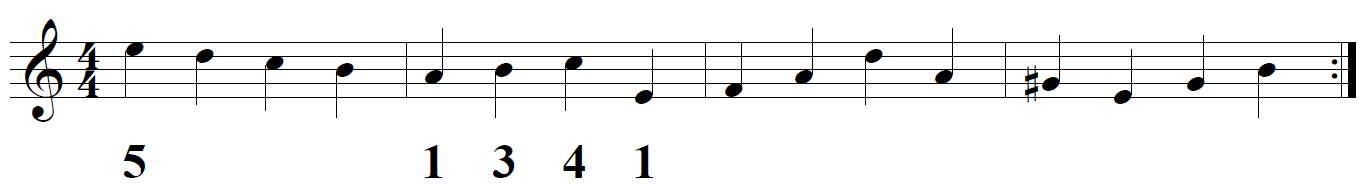
Melody 1: M IOI R-1v3

516.41 0.96


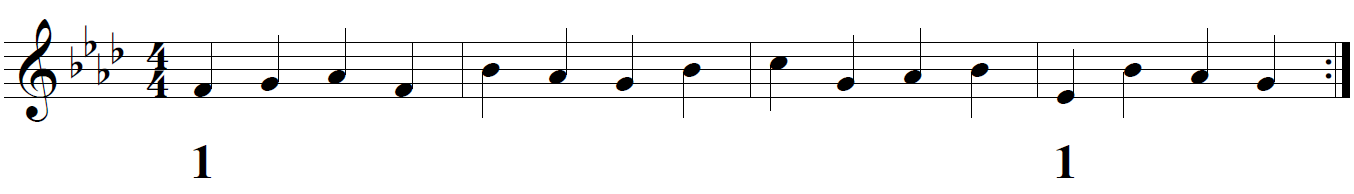
Melody 2:

493.75 0.95

Melody 3:


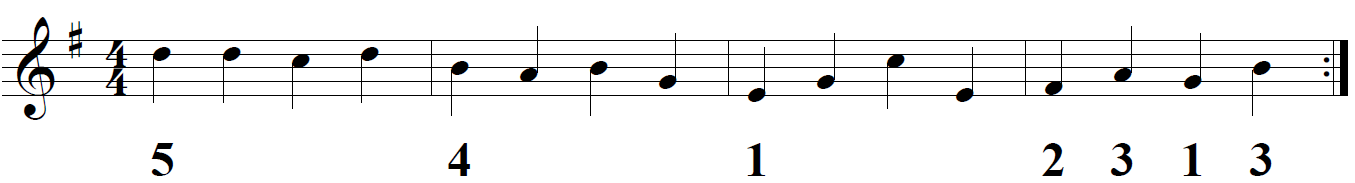


491.82 0.98

Melody 4:


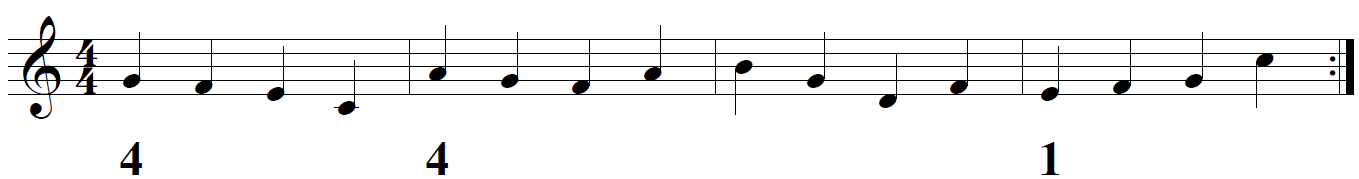


546.21 0.95

Screening Melody:


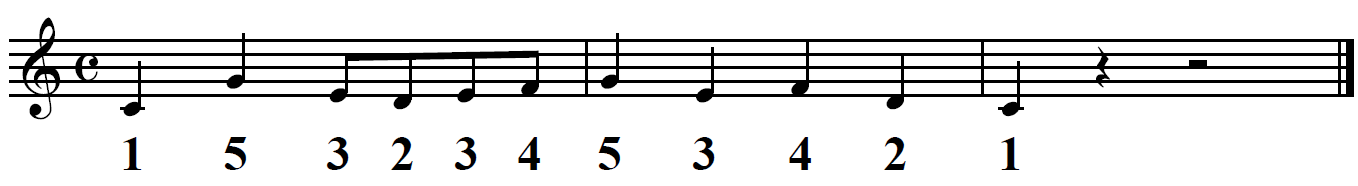


*Note:* M = mean spontaneous rate (ms) across trials and participants, R-1v3 = correlation of M IOI between the first and third repetitions of a melody across participants. All correlations *p* < .01.

Appendix C

**Phrase Stimuli in Experiment 2**

| Phrase (cyclical production) | M | R-1v3 |
| --- | --- | --- |
| …cakes are good until tomorrow… | 227.44 | 0.89 |
| …to choose between the final two… | 218.00 | 0.97 |
| …to convince her to change her plans… | 214.37 | 0.88 |
| …the queen continued to struggle… | 232.52 | 0.93 |
|  |  |  |

*Note:* M = mean spontaneous rate (ms) across trials and participants, R-1v3 = correlation of M IOI between the first and third repetitions of a sentence across participants. All correlations *p* < .01.Appendix D

**Melody Stimuli in Experiment 2**


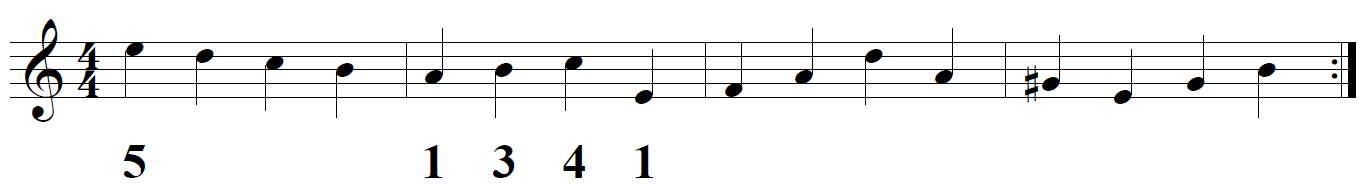
Melody 1: M IOI R-1v3

379.24 0.92

Melody 2:

363.64 0.98

Melody 3:


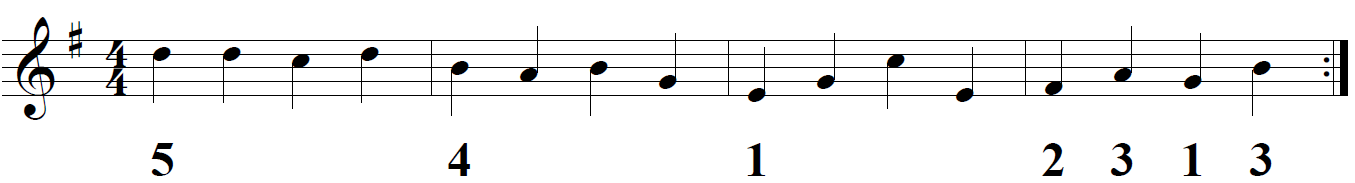


367.66 0.96

Melody 4:

402.30 0.88

*Note:* M = mean spontaneous rate (ms) across trials and participants, R-1v3 = correlation of M IOI between the first and third repetitions of a melody across participants. All correlations *p* < .01.
